# A KRT17/GATA6 hybrid state marks unique vulnerability to MYC in pancreatic cancer

**DOI:** 10.21203/rs.3.rs-11519779/v1

**Published:** 2026-11-01

**Authors:** Lyanne Delgado, Andrew Resnick, Jonathan Ogata, Jorge Villar-Samaniego, Qian Lin, Michael Horowitz, Thomas Beggs, Tristan Kwai, Anastasia Terbaci, Kayla Schwartz, Jeremy Loffredo, Katlin Wilson, Nicholas W. Bateman, Kelly A. Conrads, Jacky D. Lin, Anika Porwal, Fjord Deda, Bo Chen, Sruthi Babu, Natalia Marchenko, Emanuel F. Petricoin, Thomas P. Conrads, Scott Powers, Luisa Escobar-Hoyos, Kenneth Shroyer

**Affiliations:** Stony Brook University; Stony Brook University; Henry M. Jackson Foundation; Stony Brook University; Stony Brook University; Stony Brook University; Stony Brook University; Stony Brook University; Stony Brook University; Stony Brook University; Henry M. Jackson Foundation; Henry M. Jackson Foundation; Henry M. Jackson Foundation; Henry M. Jackson Foundation; Stony Brook University; Stony Brook University; Stony Brook University; Stony Brook University; Stony Brook University; Stony Brook University; George Mason University; Henry M. Jackson Foundation; Stony Brook University; Yale University; Stony Brook University

**Keywords:** Pancreatic cancer, cellular plasticity, KRT17, GATA6, MYC, Transitional state

## Abstract

Pancreatic ductal adenocarcinoma (PDAC) progression is driven by a poorly understood continuum of plastic malignant cell states between the “classical” (GATA6^+^) and “basal-like” (KRT17^+^) subtypes. Here, *we* integrate *scRNA-seq* with quantitative proteomics from laser-capture microdissected patient tissue and targeted mechanistic perturbations to establish a framework that simplifies this continuum and defines malignant programs linked to tumor progression, metastasis, and therapy resistance. We identify a transcriptionally and proteomically distinct KRT17^+^/GATA6^+^ hybrid cell state. Spatial analyses show that hybrid cells are enriched among de-differentiating populations, concentrate at the invasive front of primary tumors, and are significantly overrepresented in lethal liver and lung metastases, indicating that this plastic phenotype is selected during the metastatic cascade. Proteomic deconstruction reveals that the hybrid state is governed by a MYC-driven oncogenic program coupled to a metabolic shift toward oxidative phosphorylation. By individually reducing KRT17 and GATA6 expression, we establish a direct KRT17→c-MYC regulatory axis that promotes broad resistance to standard therapies while creating a specific, targetable vulnerability to MYC inhibition. Together, these findings identify the KRT17^+^/GATA6^+^ hybrid phenotype as a key node of PDAC aggressiveness and therapeutic resistance and support the use of KRT17/GATA6 as biomarkers to stratify patients for clinical trials of MYC inhibitors.

## Introduction

Pancreatic ductal adenocarcinoma (PDAC) is a highly lethal malignancy with a 5-year survival rate below 13%^1,2^ and is projected to become the second leading cause of cancer-related death in the United States by 2030^3,4^. Poor outcomes reflect late diagnosis, early dissemination, and limited durable responses to standard regimens such as FOLFIRINOX and gemcitabine/nab-paclitaxel^5^. A major barrier to therapeutic progress is extensive cellular heterogeneity within PDAC^6^, which enables subpopulations of malignant cells to survive therapy and drive recurrence and metastasis.

While genetic alterations contribute to treatment failure, resistance can also arise through non-genetic, reversible changes in malignant cell state. In PDAC, therapy can enrich drug-tolerant “persister” cells that persist through epigenetic and transcriptional adaptation^7,8^, consistent with a broader plasticity program that allows cells to transition between phenotypes with distinct invasive and therapeutic properties.

Transcriptome profiling has traditionally classified PDAC into “classical” (GATA6^+^) and “basal-like” (KRT17^+^) subtypes^9-11^. However, single-cell and spatial studies reveal that tumors often contain a continuum of states, including hybrid cells co-expressing classical and basal markers^12^. The factors that sustain these transitional states, their spatial organization within tumors, and their contributions to invasion and metastasis remain incompletely understood.

Here, we use a clinically tractable GATA6/KRT17 framework to resolve PDAC plasticity into classical-like (GATA6^+^), basal-like (KRT17^+^), and hybrid (GATA6^+^/KRT17^+^) malignant states^13-15^. By integrating *scRNA-seq*, quantitative proteomics from laser-capture microdissected patient tissue, and mechanistic perturbation of KRT17 and GATA6, we define the molecular features and tumor-localization of the hybrid state and identify the oncogenic programs that support it. These findings clarify how plastic malignant states contribute to PDAC progression and provide a rationale for biomarker-guided, plasticity-directed therapeutic strategies.

## Results

### Keratin 17 and GATA6 expression define four malignant transcriptomic programs

To characterize inter- and intra-tumoral heterogeneity in PDAC, we curated an integrated *scRNA-seq* dataset of 77,155 epithelial cells from 42 tumors across two published studies^6,16^ (Fig. 1A-B; Supplementary Table 1). Unsupervised clustering identified a distinct epithelial compartment (Fig. 1C). We then used inferred copy number variation (CNV) scores to separate malignant epithelial cells from benign counterparts (Fig. 1D) and confirmed broad expression of the pan-epithelial marker KRT19 within malignant cells (Fig. 1E).

Across malignant cells, the dominant axis of transcriptional variation was the largely reciprocal expression of KRT17 and GATA6 (Fig. 1F-G). This marker pair optimally partitioned malignant heterogeneity compared with other canonical subtype markers (Supplementary Fig. 1), motivating a supervised classification of malignant cells into four archetypal states (Fig. 1H): Classical-like (KRT17^−^/GATA6^+^; 34%), Basal-like (KRT17^+^/GATA6^−^; 28%), Hybrid (KRT17^+^/GATA6^+^; 22%), and Double-Negative (KRT17^−^/GATA6^−^; 16%). These groups showed the expected separation in KRT17 and GATA6 expressions (Fig. 1I). Evaluation using established subtype-associated genes supported the Classical-like, Basal-like, and Hybrid assignments, whereas Double-Negative cells were not captured by any single marker, consistent with a distinct transcriptional program (Fig. 1J).

We next tested whether this two-marker framework captured biologically meaningful states. The relative abundance of the four states across tumors correlated with established bulk transcriptomic subtype classifications (Supplementary Fig. 2). Gene set enrichment analysis (GSEA) further identified state-specific functional programs: Classical-like cells were enriched for differentiation, Basal-like cells for inflammatory signaling, and hybrid cells for cellular stress pathways. Double-Negative cells showed depletion of inflammatory pathways, suggesting an immunologically quiescent phenotype (Supplementary Fig. 3).

Finally, we modeled relationships among the four states. Principal component analysis placed Classical-like and Basal-like cells at opposing ends of the expression space, with hybrid cells positioned between them (Fig. 1K). Hierarchical clustering similarly separated Classical-like cells as the most distinct group and placed Double-Negative cells closest to the Basal-like population (Fig. 1L). To quantify transcriptional connectivity without implying directionality, partition-based graph abstraction (PAGA) revealed strong connectivity among states and positioned the Hybrid state as a central bridge linking Classical-like and Basal-like programs (Fig. 1M). Together, these analyses define a robust KRT17/GATA6-based framework that simplifies PDAC malignant cell-state heterogeneity and identifies a hybrid transcriptional

### Spatial analysis links tumor de-differentiation to enrichment of Hybrid and Basal-like states

To assess the spatial organization of the four KRT17/GATA6-defined malignant states, we developed and validated a multiplex immunohistochemistry (*mIHC*) assay for KRT17 and GATA6 (antibody details in Supplementary Table 6). In an independent 24-case validation cohort (Supplementary Table 2), the relative proportions of Classical-Like, Basal-Like, Hybrid, and double-negative cells measured by *mIHC* were comparable to those observed in the *scRNA-seq* dataset, supporting *mIHC* as a spatial proxy for state assignment (Supplementary Fig. 4).

We next applied this assay to a separate cohort of 40 FFPE PDAC specimens (Supplementary Table 3) to test how these states distribute across histologic differentiation. Individual glandular structures were graded as G1 (well-differentiated), G2 (intermediate), or G3 (poorly differentiated) based on architectural features and cytologic atypia (workflow in Fig. 2A).

Across 18,415 malignant epithelial cells, state composition differed systematically with gland grade (Fig. 2B). Well-differentiated (G1) glands were overwhelmingly dominated by Classical-like (KRT17^−^/GATA6^+^) cells, which comprised a median of 83% of the population. This dominance eroded with dedifferentiation, as the Classical-like fraction plummeted to just 23% in poorly-differentiated (G3) glands (p < 0.0001). Conversely, the KRT17-expressing populations, Basal-like and Hybrid, dramatically expanded to fill this void. The Basal-like (KRT17^+^/GATA6^−^) state, a minor 6% component in G1, became the predominant population in G3 glands, constituting 44% of cells (p < 0.01). Similarly, the Hybrid (KRT17^+^/GATA6^+^) population expanded from 4% in G1 to become a major component of intermediate G2 glands at 27% (p < 0.001), consistent with its role as a transitional phenotype. In stark contrast to these dynamic shifts, the Double-Negative (KRT17^−^/GATA6^−^) population remained a minor and relatively stable component, fluctuating only from 7% in G1 to 12% in G3 glands. Representative mIHC images illustrating these compositional differences are shown in Fig. 2C..

Together, these spatial data show that poorer differentiation is associated with a shift away from Classical-like cells and toward Basal-like and Hybrid phenotypes. The enrichment of KRT17^+^/GATA6^+^ Hybrid cells in poorly differentiated glandular regions motivated our subsequent analyses of the molecular programs that establish and maintain this state.

### KRT17/GATA6 state composition is associated with local invasion and metastatic disease

To extend our spatial analyses beyond glandular differentiation, we next asked whether KRT17/GATA6-defined malignant states are redistributed during local invasion. Using *mIHC* in a 10-case cohort (Supplementary Table 4), we observed a recurrent spatial pattern in which GATA6-high regions were enriched in the tumor core, whereas KRT17-high regions were prominent at the pathologist-annotated invasive front (Supplementary Fig. 6). Quantification across anatomical compartments showed that both Basal-like and Hybrid cells were significantly enriched at the invasive front relative to the tumor core (Supplementary Fig. 5B), linking these states to locally invasive tumor architecture.

We then asked whether this enrichment is also observed during systemic spread. We analyzed 25 matched primary tumors and lethal metastases from the University of Nebraska Medical Center (UNMC) Rapid Autopsy Program (Supplementary Table 5; workflow in Fig. 3A). Our analysis revealed a consistent shift in cellular composition from the primary tumor to the metastatic site (Fig. 3B; Supplementary Fig. 7). The median proportion of Basal-like (KRT17^+^/GATA6^−^) cells increased from 33.0% in primary tumors to 58.6% in lung metastases, establishing it as the dominant metastatic population. Conversely, the Classical-like (KRT17^−^/GATA6^+^) population was nearly eliminated, decreasing from 6.5% to 2.6% over the same transition. The Hybrid (KRT17^+^/GATA6^+^) state, while decreasing proportionally from 35.6% to 21.8%, remained a substantial component of the metastatic lesion. Representative images illustrate these differences (Fig. 3C). Consistent with this pattern, comparing primary tumors from diagnosis to those at time of death revealed a significant enrichment of Hybrid (KRT17^+^/GATA6^+^) cells (p = 0.0015) and a corresponding trend toward a decrease in Classical-like (KRT17^−^/GATA6^+^) cells (p = 0.0667) (Supplementary Fig. 8).

Together, these data indicate that Basal-like and Hybrid phenotypes are consistently overrepresented at sites of invasion and in lethal metastases, supporting the idea that these states are associated with aggressive disease behavior and may be favored during progression. This spatial enrichment prompted us to define the molecular programs that characterize the Hybrid state and to test its functional contributions to therapeutic resistance and metastatic fitness.

### Proteomic and functional analyses identify a MYC-linked Hybrid program with therapeutic vulnerabilities

Our *scRNA-seq* analyses positioned Classical-like and Basal-like malignant programs at opposite ends of the expression space and identified strong transcriptional connectivity through the Hybrid state. Consistent with this structure, gene set enrichment analysis across the Classical-like → Hybrid → Basal-like axis showed increasing enrichment of MYC-associated signatures (Supplementary Fig. 9), nominating MYC as a candidate regulator of Hybrid-associated biology.

To directly define protein-level programs associated with each KRT17/GATA6 state, we performed deep-scale proteomic profiling of spatially distinct malignant populations isolated from patient tissue by IHC-guided laser capture microdissection (LCM) using triplex staining for KRT17, GATA6, and PanCK (Fig. 4A, Supplementary Fig. 11A). Proteomic centroid analysis placed Hybrid (KRT17^+^/GATA6^+^) cells between Classical-like and Basal-like populations while also showing substantial orthogonal separation, consistent with a bona fide intermediate state that is proteomically distinct rather than a simple mixture of the two extremes (Fig. 4B; Supplementary Fig. 10). An Upset analysis highlighted state-restricted protein features (Supplementary Fig. 10), and MALDI imaging mass spectrometry further supported metabolic differences associated with the Hybrid program (Supplementary Fig. 13). Notably, the Double-Negative state localized proteomically closer to Classical-like cells (Supplementary Fig. 11E), supporting a non-linear organization of states rather than a single strictly linear continuum. Pathway analyses indicated that each state exhibited a characteristic proteomic program, with Hybrid cells enriched for immune-related, stress response, and cell adhesion pathways (Fig. 4C-D).

To identify upstream regulators of the Hybrid proteomic signature, transcriptional regulator analysis predicted a network of stress- and oncogenesis-associated factors (Supplementary Fig. 12), with MYC emerging as a prominent predicted regulator. Computational inference further suggested that this MYC-linked program would be associated with reduced sensitivity to standard cytotoxic therapy (Fig. 4E).

We next tested these predictions experimentally. In patient-derived organoids, we observed an inverse relationship between GATA6 and the KRT17/c-MYC axis, with low GATA6 corresponding to higher KRT17 and c-MYC protein levels (Fig. 4H). To perturb the Hybrid state in a controlled model, we used BxPC3 cells, which endogenously express both KRT17 and GATA6. Consistent with the KRT17/GATA6 framework, CRISPR-mediated loss of KRT17 shifted cells toward a more Classical-like profile, whereas siRNA-mediated reduction of GATA6 shifted cells toward a more Basal-like profile (Fig. 4F-G). Importantly, loss of KRT17 reduced c-MYC protein abundance, supporting a KRT17→c-MYC regulatory relationship (Fig. 4G).

Finally, we evaluated the therapeutic implications of this Hybrid-associated program. In cell viability assays, Hybrid (KRT17^+^/GATA6^+^) cells showed increased resistance to 5-FU (Fig. 4I), consistent with the computational prediction (Fig. 4E) and with survival associations in patient datasets (Supplementary Fig. 14). Given the convergent evidence implicating MYC, we asked whether this resistance was coupled to a targetable dependency. We therefore tested MYCi975, a small-molecule MYC inhibitor with reported preclinical activity across multiple cancer models, including triple-negative breast cancer, prostate cancer, and head and neck squamous cell carcinoma. Hybrid cells were markedly sensitive to MYCi975 (Fig. 4J), indicating that the same program linked to chemotherapy resistance can expose a pharmacologic vulnerability. Together, these multi-omic and functional data support a model in which a MYC-linked Hybrid program is a central node connecting classical-like and basal-like malignant states and is associated with aggressive biology and therapy resistance. Importantly, these results suggest that KRT17/GATA6-based stratification may identify tumors with increased susceptibility to direct MYC inhibition.

## Discussion

Translating the complex biology of pancreatic ductal adenocarcinoma (PDAC) subtypes into clinically useful biomarkers remains a central challenge. A key unmet need is to convert high-dimensional subtype biology into assays that are analytically robust, interpretable on routine specimens, and scalable for clinical validation^5,8^. Here, we address this need by developing and validating a simplified KRT17/GATA6 multiplex immunohistochemistry (*mIHC*) framework that moves beyond a static classical-basal binary classification^10,11,14^. This framework resolves malignant epithelial heterogeneity into four archetypal states and enables spatial quantification of how state composition associates with tumor dedifferentiation, invasion, metastasis, and therapy response. Importantly, these associations were supported by systematic quantification across independent cohorts, rather than representative images alone.

A central contribution of this study is orthogonal validation of these malignant programs across modalities. While *scRNA-seq* supports a continuum between classical-like (GATA6^+^) and basal-like (KRT17^+^) programs, triplex *IHC*-guided laser-capture microdissection (KRT17/GATA6/PanCK) coupled with deep proteomics demonstrates that the KRT17^+^/GATA6^+^ Hybrid state is not simply an admixture of the endpoints. Instead, Hybrid cells exhibit a distinct proteomic program and occupy an intermediate-but-separable position, consistent with similar co-expressing populations reported in recent single-cell and multiplex imaging studies^17^. We emphasize that this four-state framework is a supervised, biologically motivated stratification of a continuous malignant landscape (implemented with explicit thresholds and robustness analyses), not a claim that malignant epithelium forms four discrete unsupervised clusters. Spatially, Hybrid cells are enriched in poorly differentiated glandular regions, concentrated at invasive fronts, and overrepresented in lethal liver and lung metastases. These cross-sectional patterns are consistent with state remodeling during progression and, in the metastatic setting, with selection or outgrowth of Hybrid-associated programs—while not establishing lineage conversion or directionality in the absence of longitudinal tracing. Accordingly, our spatial data are interpreted as compartment-associated differences in state composition and connectivity, not direct evidence of deterministic or bidirectional in vivo transitions. Future lineage-aware approaches (e.g., barcoding in patient-derived models) will be required to formally distinguish selection from conversion during metastasis.

Mechanistically, our data implicate MYC as a key regulator coupled to Hybrid biology. Proteomic deconstruction predicted MYC as a prominent upstream driver, and functional perturbations provide mechanistic support for a KRT17→c-MYC relationship, as loss of KRT17 reduced c-MYC protein abundance. In parallel, the Hybrid proteome was coupled to metabolic rewiring, including a hyper-energetic metabolic rewiring^18^, evidenced by a high intracellular energy charge, altered TCA cycle intermediates, increased oxidative phosphorylation-associated programs^19^ (Supplementary Fig. 13), consistent with a stress-tolerant state capable of supporting aggressive growth^20^ in changing microenvironments.

These findings have direct translational implications. We show that Hybrid cells exhibit reduced sensitivity to 5-FU in our models yet display a targetable dependency on MYC signaling^21^, evidenced by marked sensitivity to MYC inhibition. Consistent with these observations, the patient-level association is reflected in a significant progression-free survival analysis in the context of 5-FU therapy (Supplementary Fig. 14). Together, these results nominate KRT17/GATA6 *mIHC* as a practical biomarker strategy—compatible with FFPE tissue—to quantify plasticity in situ and stratify patients for biomarker-enriched clinical trials evaluating MYC-directed therapies (e.g., NCT06059001). More broadly, our work supports the use of subtype-linked biomarkers not only to describe tumor biology but also to inform trial design and therapeutic selection^22^. Whether MYC activation downstream of the KRT17-high program functionally alters KRAS-inhibitor sensitivity remains an important mechanistic question for future work.

Our proteomics data also clarify the KRT17^−^/GATA6^−^ Double-Negative (DN) population. Importantly, the DN compartment is not a negligible population across cohorts; revised analyses define DN as a biologically interpretable program characterized by relative depletion of inflammatory and interferon-response signatures (Supplementary Fig. 3). DN regions localized proteomically closer to the Classical-like state, arguing against a strictly linear classical→basal continuum and supporting a more complex organization of malignant states. Defining the functional role of this DN program and the extent to which microenvironmental cues shape its distribution will require spatially matched multi-omic profiling and perturbation in patient-derived systems^12,17^.

This study has limitations. The two-marker framework intentionally simplifies a complex continuum, and incorporating additional lineage and basal markers (e.g., KRT81, TP63) will refine state definitions, particularly within the DN compartment. Because intra-gland heterogeneity is intrinsic to PDAC, LCM proteomes should be interpreted as state-enriched regional profiles rather than perfectly pure populations; we mitigated potential mixing by triplex *IHC*-guided capture, exclusion of visibly mixed glands, and boundary avoidance. In addition, while our functional data support a KRT17–MYC link and identify MYC inhibition as a vulnerability in Hybrid-associated biology, broader validation in patient-derived models and larger independent cohorts will be essential to establish generalizability and to support prospective clinical implementation.

In summary, we establish a clinically tractable KRT17/GATA6 *mIHC* framework that simplifies the PDAC classical–basal continuum and identifies a transcriptionally and proteomically distinct KRT17^+^/GATA6^+^ Hybrid program enriched in aggressive disease contexts. By linking this program to a KRT17→c-MYC axis and a MYC-inhibitor vulnerability, our findings provide a biomarker-driven rationale for stratifying patients for MYC-directed therapeutic trials in PDAC.

## Methods

### Computational analysis of single-cell RNA sequencing data

An integrated *scRNA-seq* dataset of PDAC tumors was curated from two public datasets^6,16^ (**Peng et al., 2019** and **Werba et al., 2023**)^6,16^. Clinical and pathologic characteristics are summarized in Supplementary Table 1. Raw sequencing data were processed using CellRanger v7.1.0. Ambient RNA contamination was reduced using CellBender^23^, which removes background transcripts arising from cell-free RNA released by lysed or damaged cells during droplet-based library preparation. After preprocessing, quality-control filtering removed cells with < 200 detected genes or > 20% mitochondrial reads.

Downstream analyses were performed in Scanpy (v1.9.3)^24^, including normalization, log-transformation, dimensionality reduction, clustering, and cell type annotation. To distinguish malignant from benign epithelium, copy-number variation (CNV) was inferred using inferCNVpy^25^ against a reference set of normal ductal cells identified within the dataset. Batch effects were corrected using Harmony^26^, and missing values were imputed using ALRA^27^ (a biological-zero-preserving approach). Trajectory structure was assessed using partition-based graph abstraction (PAGA) and diffusion pseudotime (DPT) within Scanpy, using the GATA6^+^/KRT17^−^ classical-like population as the root.

To identify pathway-level vulnerabilities, CRISPR-Cas9 gene-dependency data were obtained from the DepMap Public 24Q4 portal (Chronos scores)^28^. Differential dependency signals were mapped to MSigDB Hallmark pathways using gene set enrichment analysis (GSEA).

### Human tissue samples and cohort descriptions

All studies involving human tissue were conducted under an approved Stony Brook Medicine Institutional Review Board protocol (IRB# 94651) and in accordance with applicable guidelines and regulations. For each cohort, hematoxylin and eosin (H&E)-stained sections were independently reviewed by two board-certified surgical pathologists to confirm diagnosis and select representative tissue blocks.

The following cohorts were analyzed:

1. **Single-cell RNA sequencing discovery cohort (computational)**: Two publicly available *scRNA-seq* datasets were integrated (Peng et al., 2019, *n* = 22; Werba et al., 2023, *n* = 20), yielding 42 tumor samples. This combined dataset was used for unsupervised clustering and to define KRT17/GATA6-based transcriptional states. Clinical/pathologic characteristics were extracted from the original publications (Supplementary Table 1).
2. *mIHC* **validation cohort**: Twenty-four FFPE primary PDAC cases were accessed retrospectively from the RSOM Biobank and used to validate the *mIHC* protocol and confirm that the tissue-based state distribution recapitulated findings from the *scRNA-seq* discovery cohort (Supplementary Table 2).
3. **Glandular differentiation cohort**: A separate cohort of 40 FFPE primary PDAC cases from the RSOM Biobank was used to quantify the proportions of the four states across glandular differentiation grades (G1–G3) (Supplementary Table 3).
4. **Local invasion cohort**: Ten PDAC cases with FFPE blocks containing clear histologic evidence of invasion from pancreas into muscularis propria and duodenum were selected from the RSOM Biobank and used exclusively for spatial analysis at the invasive front (Supplementary Table 4).
5. **Matched primary–metastasis cohort**: Twenty-five cases with matched primary PDAC and distant metastases (liver and/or lung) were obtained from the University of Nebraska Medical Center (UNMC) Rapid Autopsy Program (RAP). Tissues were harvested within 1–3 hours of death to minimize post-mortem degradation and preserve in vivo molecular features. This cohort was used to test whether specific cell states are enriched at sites of lethal disease (Supplementary Table 5).

### Multiplex immunohistochemistry (\varvec*m*\varvec*I*\varvec*H*\varvec*C*)

FFPE sections (5 μm) were stained on a Discovery Ultra automated slide stainer (Roche). Slides were baked at 60°C for 32 min, followed by automated, xylene-free deparaffinization and rehydration. Antigen retrieval was performed for 64 min at 100°C in Tris-EDTA buffer (Ventana; Cat. # 950 – 500). Endogenous peroxidase activity was blocked using Discovery inhibitor (Roche; Cat. # 760–4840) for 8 min.

Staining was performed sequentially for KRT17 (SP95, Roche), pan-cytokeratin (AE1/AE3/PCK6, Roche), and GATA6 (1:100, AF1700, Novus) using Roche Discovery chromogens. After each staining round, antibody–chromogen complexes were stripped by heating slides to 93°C for 8 min in CC2 buffer (Roche). Slides were lightly counterstained with hematoxylin. Antibody and chromogen details are provided in Supplementary Table 6.

#### \varvec*I*\varvec*H*\varvec*C* image acquisition, analysis, and quantification

Whole-slide images were acquired at 20× using an Olympus VS120 slide scanner. Quantitative image analysis was performed in QuPath (v0.4.3)^29^. Tumor regions were manually annotated by a pathologist. A random forest pixel classifier was trained to segment PanCK-positive tumor epithelium and subsequently classify tumor cells based on nuclear (GATA6) and cytoplasmic (KRT17) chromogen intensity. Thresholds for positive staining were set and validated by two pathologists blinded to other annotations. Tumor cells were classified into four phenotypes: KRT17^+^/GATA6^+^, KRT17^+^/GATA6^−^, KRT17^−^/GATA6^+^, and KRT17^−^/GATA6^−^.

For the glandular differentiation cohort, individual glands were manually annotated and graded as G1 (well differentiated), G2 (moderately differentiated), or G3 (poorly differentiated/infiltrative) based on architectural complexity and cytologic atypia. State proportions were calculated within each annotated gland.

### Quantitative mass spectrometry-based proteomics of \varvec*m*\varvec*I*\varvec*H*\varvec*C*-guided laser microdissected tissue

#### \varvec*m*\varvec*I*\varvec*H*\varvec*C*

Tumor cells were isolated from 10 μm FFPE sections cut from tissue microarrays (TMAs) containing unique 4 mm^2^ cores from 13 unique patient tumors. Sections were stained with a triplex IHC panel (GATA6, KRT17, PanCK) to guide cell selection. Using a Leica LMD6 laser microdissection system, microdissected samples were collected by targeting ~ 100 cells per sample for each of the four defined states: Classical-like (KRT17^−^/GATA6^+^), Hybrid (KRT17^+^/GATA6^+^), Basal-like (KRT17^+^/GATA6^−^), and Double-Negative (KRT17^−^/GATA6^−^). Cells were collected into 20 μL of 25 mM ammonium bicarbonate (AmBic)/10% acetonitrile (ACN), pH 8.0, in MicroTubes (Pressure BioSciences).

Microdissected samples were digested using a heat-stable trypsin (0.1 μg; SMART Trypsin, Thermo Fisher Scientific) with pressure cycling technology (2320EXT barocycler, Pressure BioSciences). Peptides were dried by vacuum centrifugation and resuspended in 11 μL of 25 mM AmBic/0.015% n-dodecyl β-D-maltoside (DDM). Using an Opentrons Flex platform, peptides were loaded onto Evotips (Evosep) activated with isopropanol and equilibrated with 0.1% formic acid in water, then desalted with 0.1% formic acid in LC–MS grade water.

Peptides were analyzed using a 40 samples/day method on an EvoSep HPLC system coupled online to a timsTOF Ultra 2 mass spectrometer (Bruker Daltonik). Data were acquired by data-independent acquisition in PASEF mode (Parallel Accumulation–Serial Fragmentation), with an ion mobility range of 0.64–1.37 V·s/cm^2^ and eight PASEF ramps per cycle. Three MS/MS windows were collected per ramp starting at *m/z*400, 600, and 800, each with a 25 Th width and shifted by 25 Th for successive ramps.

### Proteomic data processing

Post-capture quality control of proteomic data was performed to validate sample identity. First, all samples were confirmed to have high enrichment of epithelial markers (e.g., EPCAM, KRT8, KRT18) and depletion of stromal (e.g., VIM). Second, the proteomic abundance of KRT17 and GATA6 was used to confirm the identity of each capture. Samples designated as KRT17-positive showed significantly higher KRT17 protein levels, and GATA6-positive samples showed significantly higher GATA6 protein levels compared to their negative counterparts. Samples that failed these QC checks were excluded from downstream analysis.

Peptide identifications were generated by searching raw files in Spectronaut v20.235 against a UniProtKB/Swiss-Prot database (Homo sapiens; downloaded May 9, 2024). Default Spectronaut settings were used with the following modifications: minimum peptide length of 6; methionine oxidation as the only variable modification; and removal of carbamidomethylation as a fixed modification. Only precursors passing FDR < 0.01 were retained.

Spectronaut outputs were imported into R (v4.4.1) using the arrow package (v16.1.0). The Spectronaut fields PEP_Stripped.Sequence, PG_ProteinAccessions, and EG_TotalQuantity_(Settings) were extracted as peptide sequence, mapped protein accessions, and precursor MS2 intensity, respectively. Precursor sequences were mapped back to the reference database; precursors mapping to multiple proteins were assigned to the protein supported by the largest number of unique peptides. Protein-level abundances were calculated using MaxLFQ with up to 10 representative precursors selected based on coverage and intensity. Initial data processing revealed high variability in proteomic depth across samples, with protein identifications per sample ranging from < 500 to > 5,000. To ensure robust downstream analysis, a technical quality control filter was applied. Samples with ≥ 50% missing protein measurements were excluded. This filtering step resulted in the retention of 46 high-quality samples distributed across the four states as follows: Hybrid (n = 17), Basal-like (n = 12), Double-Negative (n = 9), and Classical-like (n = 8). Following sample filtering, proteins observed in ≤ 50% of the remaining samples were removed, and residual missing values were imputed by k-nearest neighbors (k = 11; impute package v1.72.0). Protein abundances were median-normalized per sample and log2-transformed.

### Proteomics differential analyses

Differential protein-abundance analyses were performed in R (v4.4.1) using limma (v3.60.4). Design matrices were specified without an intercept, using cell population as the only covariate. *P* values were adjusted by the Benjamini–Hochberg method. Pairwise comparisons were performed across all cell populations.

To construct cell-population-associated protein sets, proteins meeting a nominal limma *P* < 0.01 were assigned according to the sign of the estimated log fold change (positive to the numerator population; negative to the denominator population). Assignments were aggregated across comparisons and deduplicated within each population. Assignments to pooled “All_others” categories from one-versus-rest contrasts were excluded. The union of proteins assigned to at least one population was used to generate a binary membership matrix indicating assignment to limma-derived protein sets (assignment reflects differential enrichment and does not imply strict detection/absence in the original search). Intersections among sets were visualized using ComplexUpset (v1.3.3). Intersections involving ≥ 2 populations and containing ≥ 3 proteins were displayed; empty combinations were retained. Sets were ordered by decreasing size.

### Principal-component centroid projection analysis of the Hybrid state

A principal-component centroid projection analysis was used to test whether the GATA6^+^/KRT17^+^ Hybrid population occupied an intermediate position along a proteomic axis connecting the GATA6^+^/KRT17^−^ and GATA6^−^/KRT17^+^ populations. Proteins were restricted to those differentially abundant between GATA6^+^/KRT17^−^ and GATA6^−^/KRT17^+^ by limma at nominal *P* < 0.01. Principal component analysis (PCA) was performed on centered and unit-variance-scaled protein abundances.

For each population, centroids were defined as the mean PCA scores across samples in that population. The reference axis was defined as the vector from the GATA6^+^/KRT17^−^ centroid $C_{\text{GATA}6}$ to the GATA6^−^/KRT17^+^ centroid $C_{\text{KRT}17}$. For a population centroid $C_{T}$, its relative projection P onto the reference axis was calculated as:

$$P=\frac{\left(C_{T}-C_{\text{GATA}6})\cdot (C_{\text{KRT}17}-C_{\text{GATA}6}\right)}{{\left\|C_{\text{KRT}17}-C_{\text{GATA}6}\right\|}^{2}}.$$

Here, P=0 corresponds to $C_{\text{GATA}6}$ and P=1 corresponds to $C_{\text{KRT}17}$. Values between 0 and 1 indicate a position between endpoints, whereas values outside this range indicate projection beyond an endpoint.

Perpendicular deviation from the axis was computed as the orthogonal distance from $C_{T}$ to the reference axis, normalized by the Euclidean distance between $C_{\text{GATA}6}$ and $C_{\text{KRT}17}$(orthogonal-distance ratio). Stability was evaluated using PCA spaces containing the first 2–10 principal components. Uncertainty was estimated with 5,000 nonparametric bootstrap iterations, resampling with replacement within each population. Ninety-five percent confidence intervals were defined by the 2.5th and 97.5th percentiles of the bootstrap distribution (Supplementary Fig. 11).

### Ingenuity Pathway Analysis

Upstream regulator analyses, including the 5-fluorouracil inhibition signature, were performed in QIAGEN Ingenuity Pathway Analysis (IPA) by importing limma outputs (Gene.Symbol, logFC, and P.Value). Proteins meeting a nominal *p* < 0.05 threshold were included.

### MALDI imaging

A tissue section adjacent to those used for *mIHC* and H&E was processed for MALDI imaging. Sections were baked at 60°C, deparaffinized in fresh xylene, and sprayed with 9-aminoacridine (9-AA) matrix (5 mg/mL in 70% ethanol; Sigma-Aldrich) using an HTX M5 sprayer. Data were acquired on a timsTOF fleX mass spectrometer (Bruker Daltonics) at 20 μm spatial resolution in negative ion mode over a mass range of 50–650 *m/z*.

MALDI data were imported into SCiLS Lab (v2024b), and *mIHC* and H&E images were co-registered. Metabolites were annotated by matching *m/z* values against a predefined feature list with a tolerance of ± 10 ppm. Spatial segmentation based on KRT17 positivity was used to generate region-specific metabolic profiles.

### Cell culture and functional assays

All functional assays were performed using the BxPC-3 human PDAC cell line, which endogenously expresses both KRT17 and GATA6 (KRT17^+^/GATA6^+^). General cell culture, authentication, and quality control methods, including routine mycoplasma screening, were performed as previously described^30^.

### Genetic Perturbations

To generate a KRT17 loss-of-function model, BxPC-3 cells were electroporated with CRISPR-Cas9 ribonucleoproteins targeting KRT17 (sgRNA: 5'-CCAGTACTACAGGACAATTG-3') following the manufacturer's protocol (Synthego). GATA6 knockdown was achieved by transient transfection with a validated small interfering RNA (siRNA) targeting GATA6 (Dharmacon) using Lipofectamine RNAiMAX (Thermo Fisher Scientific). A non-targeting siRNA was used as a control. Successful perturbations were confirmed by western blot.

### Western Blotting

Cell lysates were prepared in RIPA buffer (Thermo Fisher) containing a protease and phosphatase inhibitor cocktail (Thermo Fisher Scientific). Protein concentration was measured using the BCA assay (Thermo Fisher Scientific). Equal amounts of protein (20 μg) were resolved by SDS-PAGE, transferred to PVDF membranes, and probed with primary antibodies against KRT17 (1:1000; Abcam), GATA6 (1:1000, AF1700, Novus), c-MYC (1:1000; Cell Signaling Technology #5605), and β-actin (1:5000; Sigma-Aldrich) as a loading control. Blots were imaged on a LI-COR Odyssey system.

### Drug Sensitivity Assays

Cells were plated in 96-well plates and treated 24 hours later with a concentration range of 5-fluorouracil (5-FU) or the MYC inhibitor MYCi975 (Selleckchem). Cell viability was measured 72 hours post-treatment using an MTT assay. Dose–response curves were generated, and half-maximal effective concentration (EC50) values were calculated using nonlinear regression in GraphPad Prism (v9.0).

### Statistical analysis

Statistical analyses were performed in GraphPad Prism 9 unless otherwise stated. Data are presented as mean ± SEM. Two-group comparisons used two-tailed Student’s *t*-tests or Mann-Whitney *U* tests for nonparametric data. Matched comparisons used paired Wilcoxon signed-rank tests. Multi-group comparisons (e.g., across gland grades) used one-way ANOVA with Tukey’s post hoc test. Survival curves were generated by the Kaplan–Meier method and compared using the log-rank test. *IC*_50_ values were estimated by nonlinear regression using a log(inhibitor) versus normalized response model. All *p* values were two-sided, and significance was defined as *p* ≤ 0.05.

## Supplementary Material

This is a list of supplementary files associated with this preprint. Click to download.

• SUPPLEMENTARYMATERIALS.docx

## Figures and Tables

**Figure 1:**
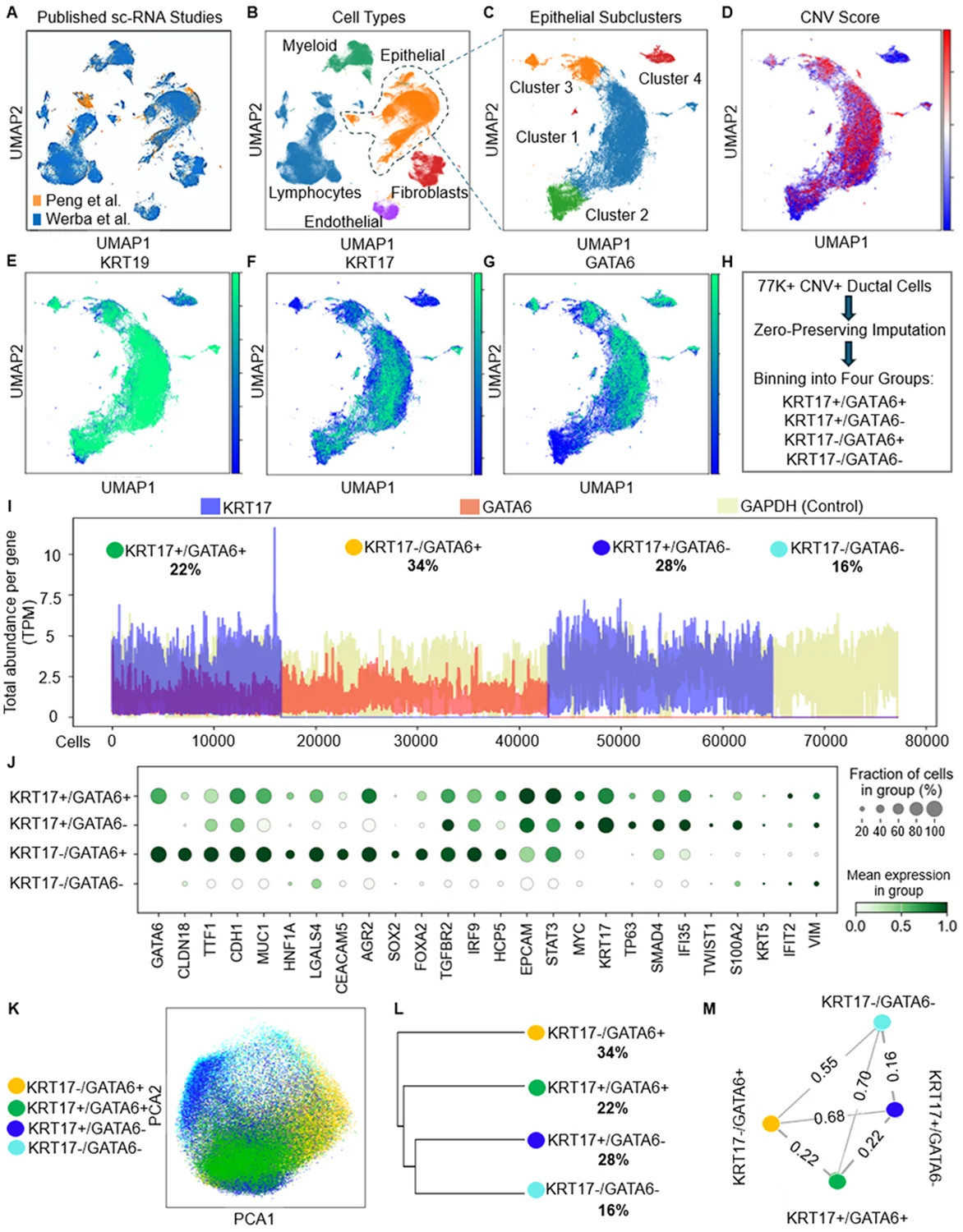
Single-cell profiling of PDAC defines four transcriptomic programs based on KRT17 and GATA6 expressions. (A) UMAP of integrated PDAC datasets from Peng et al. (n=22) and Werba et al. (n=20), with cells colored by study origin. (B) UMAP of all cells colored by major cell lineages. (C) UMAP highlighting the isolated epithelial cell compartment. (D) UMAP of epithelial cells colored by inferred copy number variation (CNV) score to distinguish malignant from non-malignant cells. (E) UMAP showing expression of the epithelial marker KRT19. (F-G) UMAPs showing expression of (F) KRT17 and (G) GATA6 across malignant cells. (H) Schematic illustrating the four expression-based categories. (I) Expression overlay plot visualizing KRT17, GATA6, and GAPDH across the four categories. (J) Dot plot showing the expression of canonical Moffitt signature genes across the four categories. (K) Principal Component Analysis (PCA) of malignant cells. (L) Hierarchical clustering dendrogram of the four categories. (M) Partition-based graph abstraction (PAGA) plot showing transcriptional connectivity between categories.

**Figure 2:**
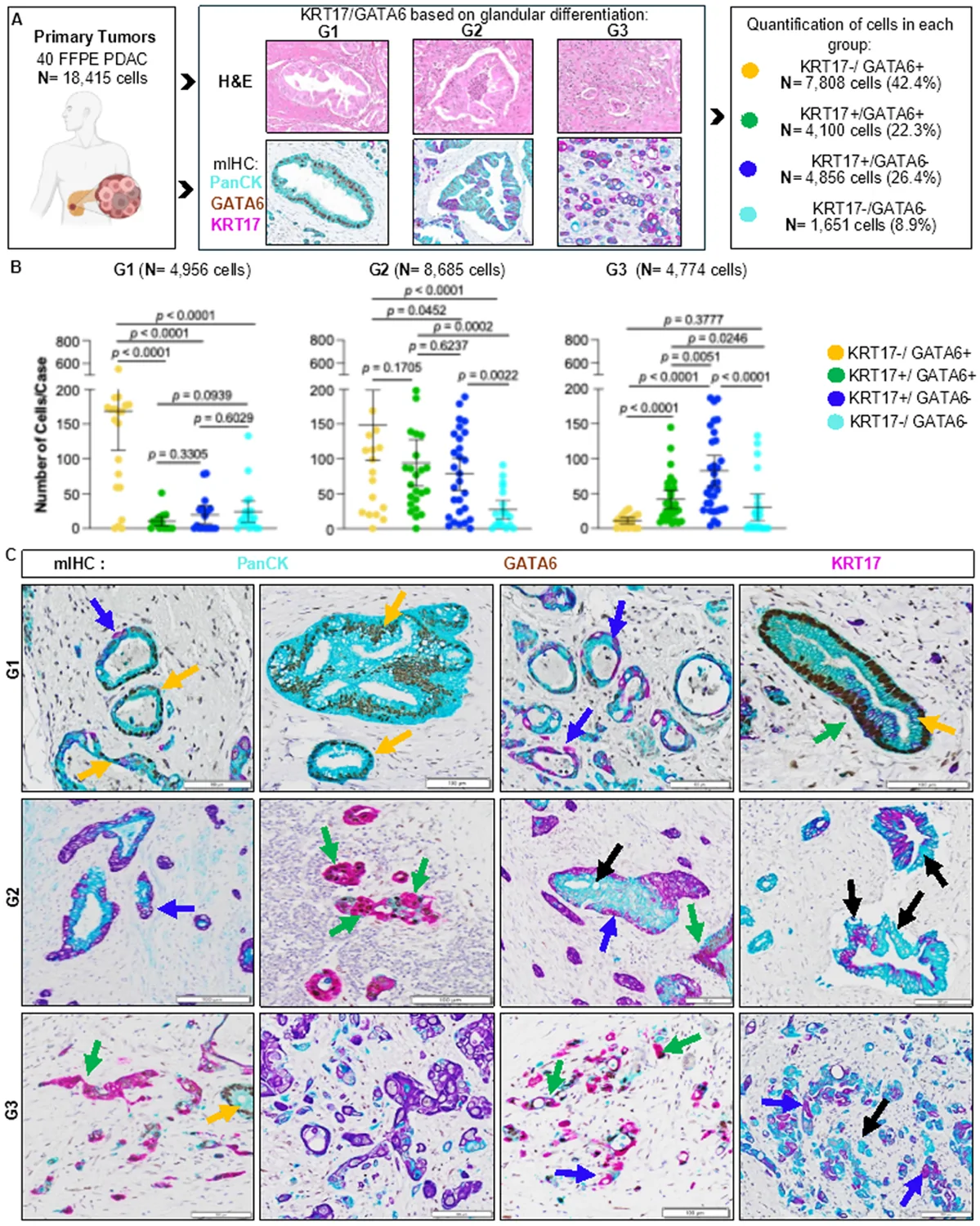
KRT17+/GATA6+ hybrid cells emerge with tumor dedifferentiation. (A) Schematic of the analysis workflow correlating glandular differentiation grade (G1-G3) with cell population abundance. (B) Quantification of the four cell populations across grades of glandular differentiation (G1-G3). (C) Representative multiplex immunohistochemistry images for each glandular grade. Stain Key: KRT17 (Purple), GATA6 (Brown), Pan-Keratin (Teal). Arrow colors indicate cell type: KRT17−/GATA6+ (yellow), KRT17+/GATA6+ (green), KRT17+/GATA6− (blue), and KRT17−/GATA6− (cyan). Scale bars: 100 μm.

**Figure 3:**
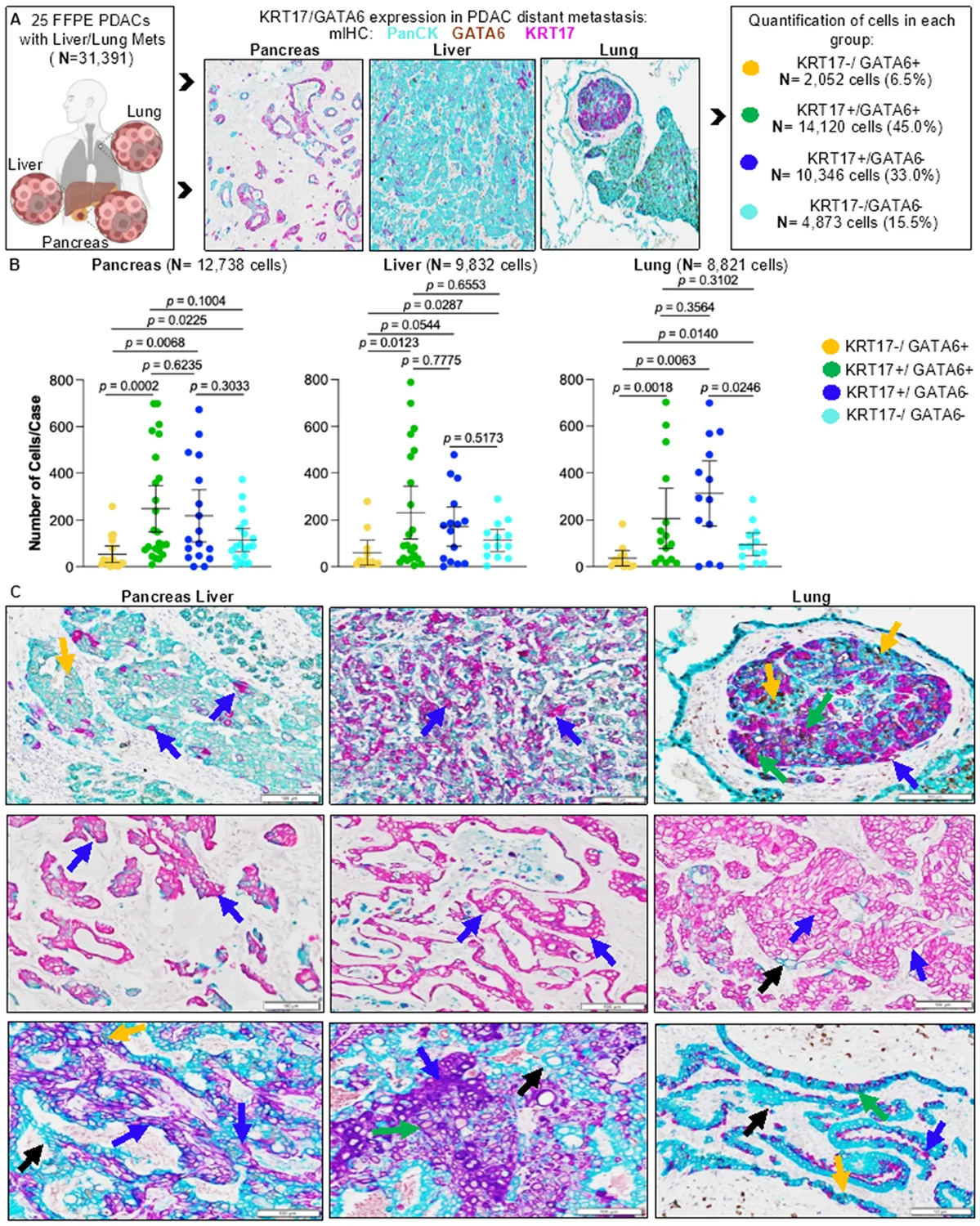
Metastases are Enriched for KRT17-Positive Tumor Cells. (A) Schematic of the workflow for comparing cell populations in 25 matched primary and metastatic tumors. (B) Quantification of the four KRT17/GATA6-defined cell populations in matched primary tumors and their corresponding metastases. (C) Representative immunohistochemistry (IHC) images of a primary tumor and a matched liver metastasis. Stain Key: KRT17 (Purple), GATA6 (Brown), Pan-Keratin (Teal). Arrow colors indicate cell type: KRT17−/GATA6+ (yellow), KRT17+/GATA6+ (green), KRT17+/GATA6− (blue), and KRT17−/GATA6− (Cyan). Scale bars: 100 μm.

**Figure 4:**
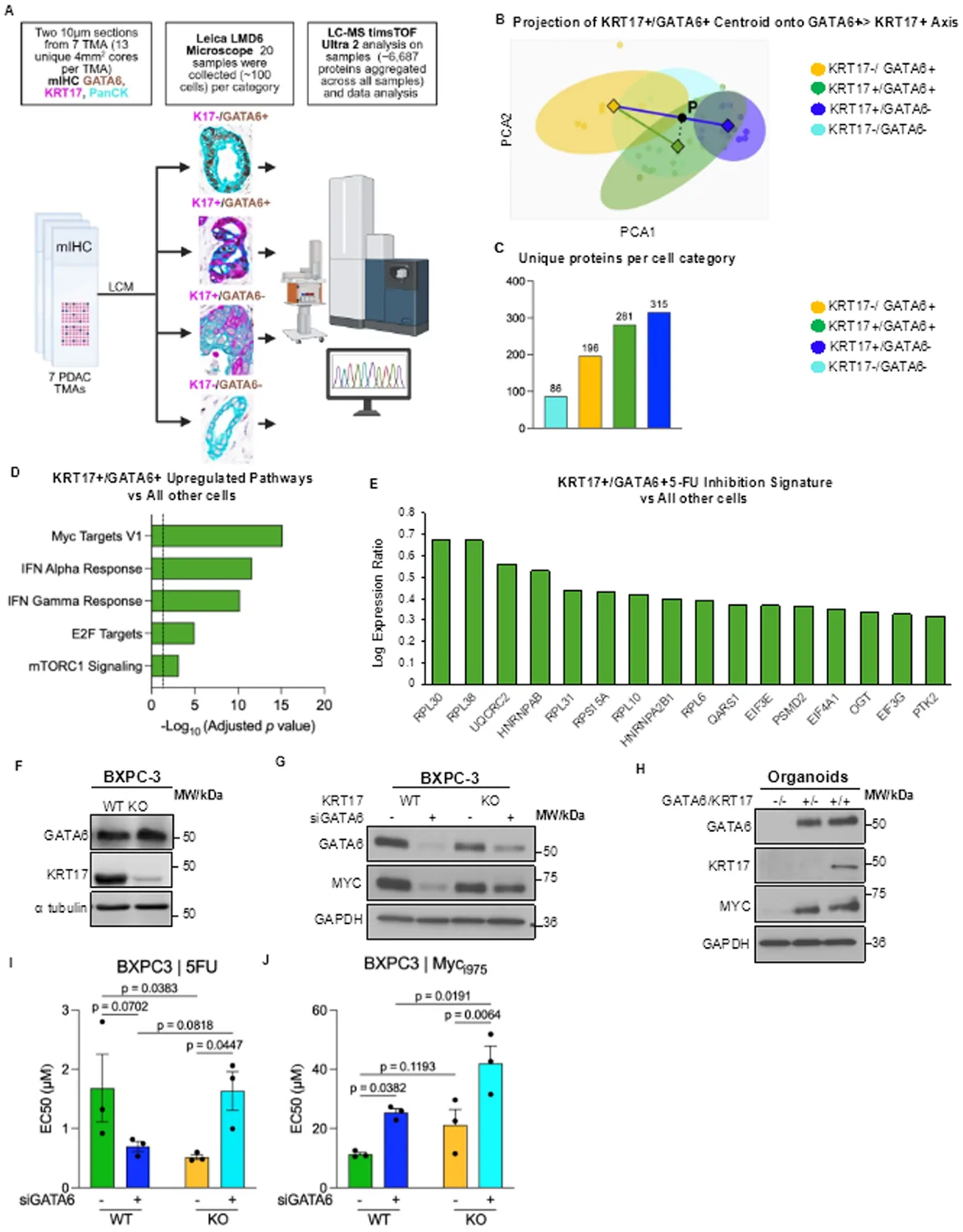
Proteomics Defines a Transitional KRT17+/GATA6+ State with a Specific Therapeutic Vulnerability. (A) Spatially-resolved proteomic workflow: (1) mIHC identification, (2) Laser Capture Microdissection (LCM), (3) LC-MS/MS profiling. (B) Centroid analysis positions the Hybrid population between the GATA6+ and KRT17+ states (projection distance = 0.61). (C) Bar graph showing the number of unique proteins per state. (D) Pathway analysis of the top five enriched biological pathways in Hybrid cells. (E) Ingenuity Pathway Analysis (IPA) predicts reduced 5-fluorouracil (5-FU) efficacy in Hybrid cells. (F-G) Western blots confirming protein expression changes in BxPC3 cells upon KRT17-KO (F) and siGATA6 (G). (H) Western blot of three PDO lines showing an inverse correlation between GATA6 and KRT17/c-MYC. (I) Cell viability assay showing Hybrid cells are resistant to 5-FU. (K) Cell viability assay demonstrating KRT17 expression creates a vulnerability to the MYC inhibitor MYCi975.

## Data Availability

The clinical data sets generated during and/or analyzed during the current study are not publicly available due to IRB regulations. All requests for data should be submitted to the corresponding authors for consideration.
